# Sex-specific clustering of metabolic risk factors and cancer risk: a longitudinal study in Iran

**DOI:** 10.1186/s13293-020-00296-6

**Published:** 2020-04-25

**Authors:** Azra Ramezankhani, Fereidoun Azizi, Farzad Hadaegh

**Affiliations:** 1 Prevention of Metabolic Disorders Research Center, Research Institute for Endocrine Sciences, Shahid Beheshti University of Medical Sciences, Yemen Street, Shahid Chamran Highway, P.O. Box: 19395-4763, Tehran, Iran; 2 Endocrine Research Center, Research Institute for Endocrine Sciences, Shahid Beheshti University of Medical Sciences, Tehran, Iran

**Keywords:** Cancer, Risk factor, Metabolic, Cluster

## Abstract

**Background:**

Cancer is a major cause of death in low- and middle-income countries. A large number of studies have shown that some of the metabolic risk factors (MRFs) tend to cluster in individuals. We examined the synergistic effects of multiple MRFs and cancer risk among Iranian adults.

**Methods:**

Among 8593 (3929 men) participants aged ≥ 30 years, the self-organizing map (SOM) was applied to clustering of four MRFs including high fasting plasma glucose (HFPG), high total cholesterol (HTC), high systolic blood pressure (HSBP), and high body mass index (HBMI). The Cox proportional hazards model was used to investigate the association between clusters with cancer incidence during a median of 14.0 years of follow-up.

**Results:**

During the study period, 265 new cases of cancer were identified among participants at risk. The incidence density rate was 2.5 per 1000 person years in total population. About 32 and 40% of men and women, respectively, had three or four MRFs. We identified seven clusters of MRFs in both men and women. In both genders, MRFs were clustered in those with older age. Further, inverse associations were found between current smoking in men, and education level and passive smoking in women and clustering of MRFs. In men, a cluster with 100% HSBP and HBMI had the highest risk for overall cancer. While, among women, a cluster with 100% HFPG and 93% HBMI yielded the highest risk for cancer. The risk was decreased when HBMI accompanied by HTC.

**Conclusions:**

Clustering patterns may reflect underlying link between MRFs and cancer and could potentially facilitate tailored health promotion interventions.

## Background

Cancer is already a major cause of death in low- and middle-income countries [1, 2]. In 2016, about 15 million new cases of cancer and 9 million deaths from cancer were reported worldwide, which has increased by 18% relative to 10 years previously [3]. In 2010, cancer was the second main cause of death in Iran [4]. According to the Global Burden of Disease Study 2016, several metabolic risk factors (MRFs) including high fasting plasma glucose (HFPG), high body mass index (HBMI), high systolic blood pressure (HSBP), and high total cholesterol (HTC) were the leading risk factors for non-communicable diseases; among them, HFPG and HBMI have been associated with increased risk of several of the more common cancers [5].

A large number of studies have repeatedly shown that some of the MRFs tend to cluster and co-occur in individuals [6, 7], and clusters of these factors may have synergistic properties, such that the combined effect of these factors is much worse than the sum of each risk factor in isolation [8]. Although growing evidence shows relation between a single MRF and risk of cancer [9–11], very little research has examined the association between clusters of MRFs and cancer incidence [8]. Due to the synergistic effects of multiple MRFs, identifying clusters of these factors can help implementing multiple interventions at the population level to reduce the risk of cancers [12]. The purpose of the present study was therefore to identify (1) distinct clusters of MRFs among Iranian adults, (2) the association between identified clusters and demographic, social, and behavioral characteristics, and (3) the relation between different clusters of MRFs and incidence of cancers.

We applied the self-organizing map (SOM) [13] to identify different clusters of four MRFs including HFPG, HTC, HBMI, and HSBP among participants. SOM is a kind of neural network learning without a supervisor which is used for clustering and data visualization. SOM simplify complex and multidimensional data and have been used in a broad range of fields including medicine [7, 14]. To accomplish our objectives, we analyzed data from Tehran Lipid and Glucose Study (TLGS).

## Materials and methods

### Study population

**Fig. 1:**
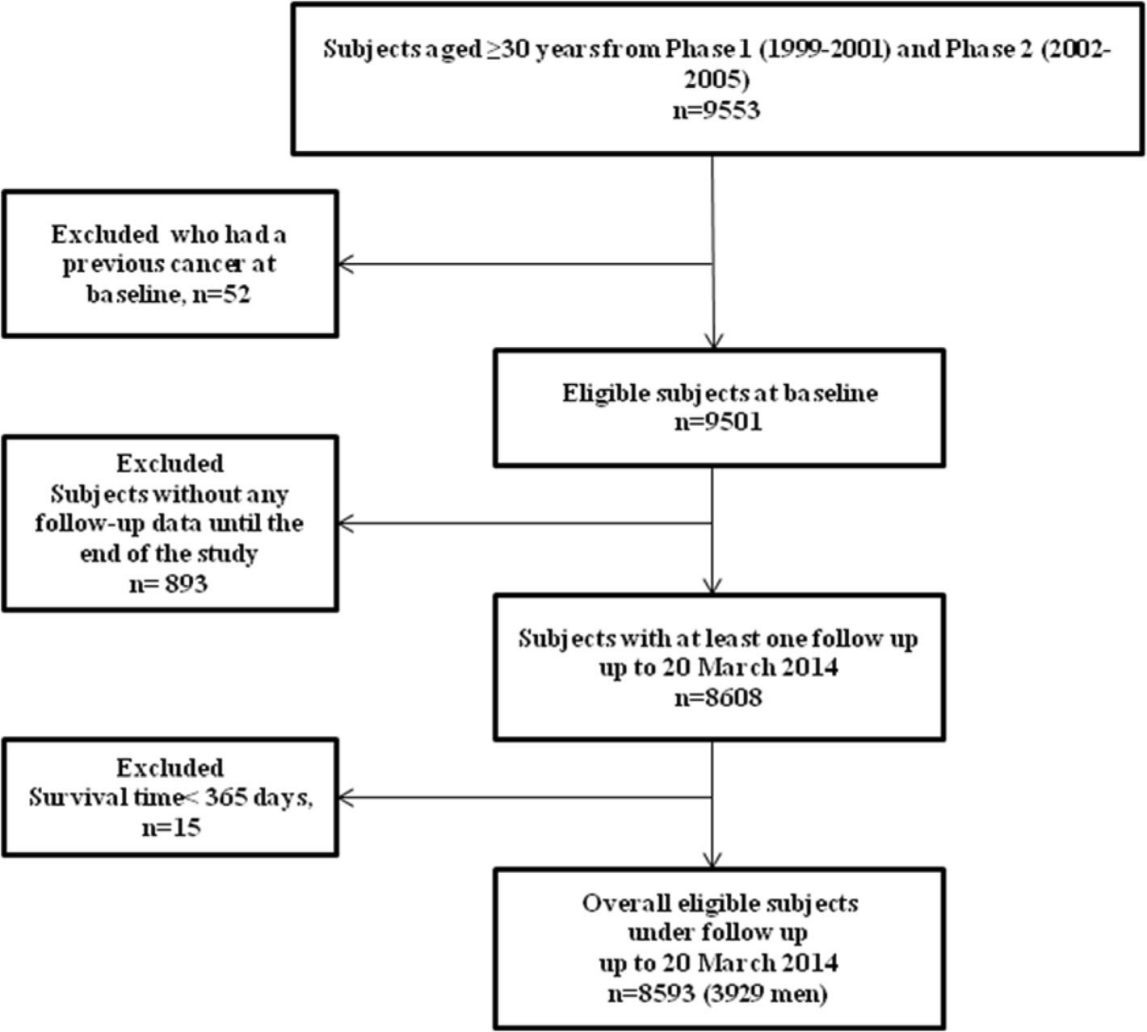
Study participant selection. Tehran Lipid and Glucose Study (1999–2014)

The TLGS is a large-scale, long-term, and community-based prospective study performed on a representative sample of residents of district-13 of Tehran. The study is described in detail elsewhere [15]. In brief, about 15,000 individuals aged ≥ 3 years participated in the first phase of the study (1999–2001) and a total of 3550 new subjects were included in phase 2 (2002–2005). Annual follow-up of the cohort for different outcomes began from entry to study until the end of the study (20 March 2014). For the current study, all subjects aged ≥ 30 years from the first and second phases (*n* = 9553) were included. Participants with previous cancer at baseline (*n* = 52), without any follow-up data until the end of the study (*n* = 893), and with survival time under 365 days (*n* = 15) were excluded. The remaining 8593 (3929 men) participants (90% of eligible sample) contributed until the censoring date, first cancer occurrence, or death from any cause (Fig. 1). The ethics committee of the Research Institute for Endocrine Sciences of Shahid Beheshti University of Medical Sciences approved the study, and informed written consent was obtained from all participants.

### Baseline data collection

Standardized interviewer-administered questionnaires were used to obtain data on demographic characteristics, smoking status, educational level, physical activity, and prescription medications. Height and weight were measured in standardized ways, and BMI was calculated as weight (kg)/height^2^ (m^2^). Sitting blood pressure (BP) was measured twice by trained technicians, at least 15 min apart, and the mean value was considered as the subject’s BP. A single blood sample was drawn following an overnight fast to determine the FPG and TC following a standardized protocol [15]. Physical activity level was assessed using the Lipid Research Clinic [16] questionnaire in the first phase of the study. It was substituted by the Modifiable Activity Questionnaire from the second phase for obtaining the quantitative measure of physical activity level [17].

### Definition of confounders

Education attainment was categorized into three groups: 0–8 years, 9–12 years, and over 12 years. Current smoker was considered as someone who is currently using cigarettes or other smoking implements daily, non-daily, and occasionally. Former smokers were defined as subjects who have smoked daily or occasionally and those who have quit smoking. Passive smokers were non-smokers who were exposed to environmental tobacco smoking. Never smokers were adults who reported not smoking any product during their entire life. In the first phase, low physical activity was defined as doing exercise or labor < three times a week, and in the second phase, it was defined as achieving a scores ≤ 600 MET (metabolic equivalent task) − minutes per week [18].

### Definitions of MRFs

Four MRFs were defined based on the well-known cut-offs [6] as follows: HBMI (BMI ≥ 25 kg/m^2^), HBP (SBP ≥ 120 mmHg), HTC (serum TC ≥ 5.18 mmol/L), and HFPG (FPG level ≥ 5.55 mmol/L).

### Follow-up and outcome classification

Subjects with no history of cancer were followed annually from baseline examinations until their first cancer diagnosis, death, emigration, or end of study (March 20, 2014), whichever occurred first. The main outcome was a composite of cancer types. Death certificates and the medical records of the hospitalized patients were used to supplement the information on cancer incidence. Only patients with pathologically proven cancer leading to hospitalization were enrolled. The collected information was then evaluated by an outcome committee consisting of an endocrinologist, an internist, a cardiologist, an epidemiologist, and other experts. The diagnoses of cancers, including histologic types, were coded according to the International Classification of Diseases 10th Revision. Time to event was defined as time of censoring or having the cancer, whichever occurred first. We censored individuals at the time of other causes of death, loss to follow-up during the study period (who had at least one successful phone contact during the study period), and being in the study until 20 March 2014 without cancer occurrence.

### Statistical methods

A chi-square goodness of fit test, independent samples *t*-test, and one-way analysis of variance (ANOVA) were used to compare the categorical and continuous variables, as appropriate. Incidence density rate of cancers was calculated by dividing the number of events by the person years at risk. Missing data (after applying the exclusion criteria) were 2.5, 1.4, 2.7, and 3.0% in men, and 3.7, 1.6, 6.0, and 5.8% in women for smoking status, BMI, TC, and physical activity level, respectively. Therefore, the multivariate imputation by chained equations (MICE) (mice package in R software) [19] was implemented for handling missing data.

The SOM was applied to identify clusters of individuals with similar patterns of four MRFs separately for men and women. SOM is a nonparametric and unsupervised learning based on a neural network technique which groups similar individuals based on multivariate distance and forms a low-dimensional map of training dataset. Typically, SOM consisted in neurons (units or cells) organized on a regular two-dimensional grid, usually represented as cells on hexagonal or rectangular lattice. Similar individuals in terms of their characteristics are placed close together on the SOM grid, while individuals far apart on the map are different from each other [13]. For assessing cluster quality, we used the Silhouette width index that provides a comparison of the tightness of the groupings of subjects within each cluster to the separation between clusters. The value of this index is between −1 and 1. Silhouette width above 0.5 is considered as a reasonable clustering [20].

The multinomial logistic regression analysis were conducted to identify which factors (i.e., age, smoking status, educational level, and physical activity level) were associated with being a member of a cluster at baseline. Finally, one categorical variables with *k* level (*k* is the number of cluster) was specified and the association between cluster membership with the cancer incidence was assessed by the Cox proportional hazards model without and with adjusting for aforementioned baseline characteristics. The proportional hazard assumption was checked using statistical tests based on the scaled Schoenfeld residuals. All models were stratified by sex. Analyses were performed in the R statistical package, v.3.4.0 (www.r-project.org) using packages kohonen [14], mice [21], survival [22], and cluster [23]. Two-tailed *P* < 0.05 was considered significant.

## Results

### Baseline characteristics

After exclusions, the study sample consisted of 8593 total individuals (3929 men and 4664 women) aged ≥ 30 years. The mean (standard deviation (SD)) age at cohort entry were 48.1 (13.1) and 46.5 (11.7) years for men and women, respectively. During a median of 14.0-year follow-up (interquartile range, 10.2–14.5 years), 265 new cases of cancer (132 in men and 133 in women) were identified among participants at risk. The incidence density rate was 2.5 (95% confidence interval (95% CI) 2.2–2.8) per 1000 person years in total population and 2.8 (2.3–3.3) and 2.3 (1.9–2.7) in men and women, respectively. Among men, the most common cancers in terms of new cases were tumors of the digestive system (40%), tumors of hematopoietic and lymphoid tissues (16%), male genital system (15%), urinary system (14%), and respiratory system (6%). Tumors of the female genital system (46%), digestive system (24%), and hematopoietic and lymphoid tissues (7%) were the three most common cancer site groups among females.

Table 1 shows the baseline characteristics of the participants. In both genders, non-cancer subjects were younger and less educated. In addition, they had lower mean of SBP in both genders. About 12.5% of men had no MRFs, and 32.3% of them had three or four MRFs. The corresponding values were 8.6 and 40.4% in women.

**Table 1:** Baseline characteristics of study participants, stratified by gender and cancer status, Tehran Lipid and Glucose Study (1999–2014)

Variables	Men			Women		
	Non-cancer ***n*** = 3797	Cancer ***n*** = 132	***P*** value	Non-cancer ***n*** = 4531	Cancer ***n*** = 133	*P value
**Age (years)**	47.8(13.0)	59.4(11.5)	< 0.001	46.3(11.6)	52.1(12.0)	<0.001
**BMI (kg/ m**^**2**^**)**	26.2(3.9)	25.9(3.9)	0.451	28.6(4.7)	28.6(5.1)	0.935
**FPG (mmol/L)**	5.5(1.7)	5.8(2.0)	0.167	5.6(2.1)	5.7(1.9)	0.668
**TC (mmol/L)**	5.3(1.1)	5.3(1.1)	0.698	5.6(1.2)	5.8(1.1)	0.063
**SBP (mmHg)**	121.4(19.1)	131.9(19.8)	< 0.001	121.4(20.4)	126.8(22.1)	0.003
**DBP (mmHg)**	78.2(11.2)	79.9(11.6)	0.083	78.8(10.8)	79.6(12.0)	0.390
**Education**						
Level 1 (0–8 years)	29.4(76.5)	99(75.0)	< 0.001	3477(76.7)	91(68.4)	0.010
Level 2 (9–11 years)	672(17.7)	12(9.1)		340(7.5)	8(6.0)	
Level 3 (> 12 years)	221(5.8)	21(15.9)		714(15.8)	34(25.6)	
**Low physical activity**	2758(72.6)	92(69.7)	0.257	3220(71.1)	93(69.9)	0.420
**Smoking**						
Never	1460(38.5)	58(43.9)	0.063	3377(74.5)	100(75.2)	0.224
Past	622(16.4)	29(22.0)		107(2.4)	6(4.5)	
Passive	487(12.8)	15(11.4)		824(18.2)	24(18.0)	
Current	1228(32.3)	30(22.7)		223(4.9)	3(2.3)	

**Fig. 2:**
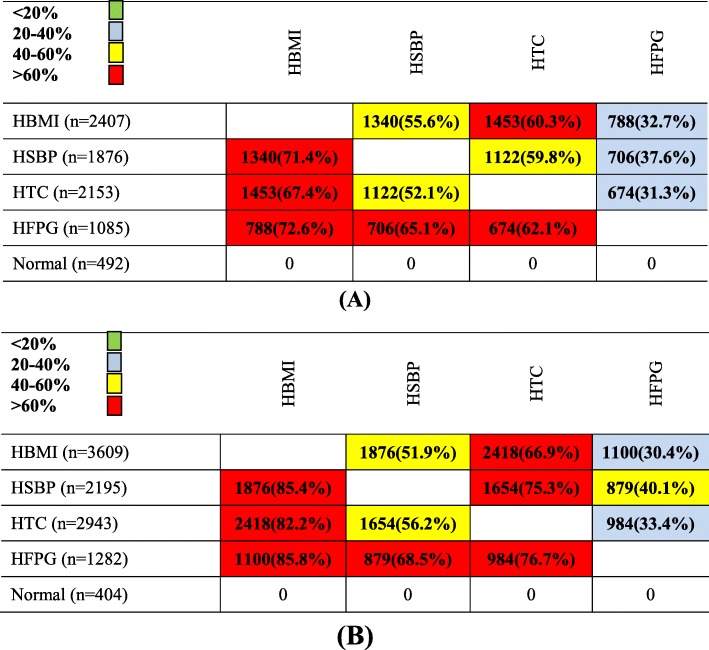
The relationship between four MRFs in the study population. **a** Male. **b** Female. First columns show the total number of population in each row, and the next columns show the number (percentage) of each MRF in each row. For easy interpretation, the table is colored: green, < 20%; blue, 20–40%; yellow, 40–60%; and red, > 60% prevalence. MRFs: metabolic risk factors; HBMI: high body mass index; HFPG: high fasting plasma glucose; HTC: high total cholesterol

The frequency and percentage of different MRFs in population by each of the four MRFs are shown in Fig. 2. For example, HBMI, HSBP, and HTC were highly prevalent (more than 60%) among men and women with HFPG (Fig. 2).

### Number of clusters

**Fig. 3:**
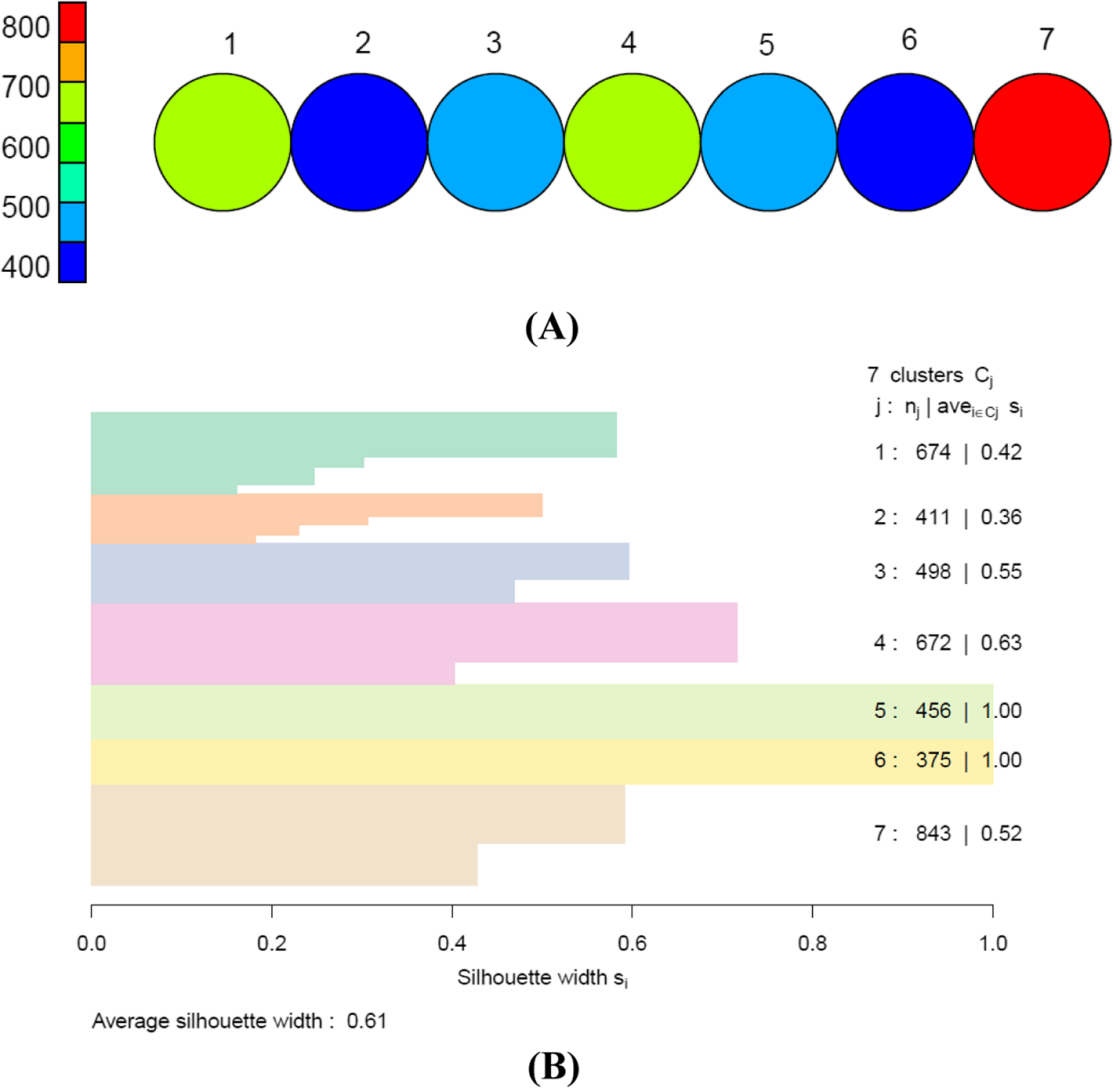
**a** Overall clusters identified by SOM in men with size of 7 × 1. Each circle represents one cluster. Each cluster contains 400–800 subjects who are extremely similar to each other regarding to four MRFs. **b** The silhouette width of each cluster and average silhouette width of all clusters. SOM: self-organizing maps; MRFs: metabolic risk factors

**Fig. 4:**
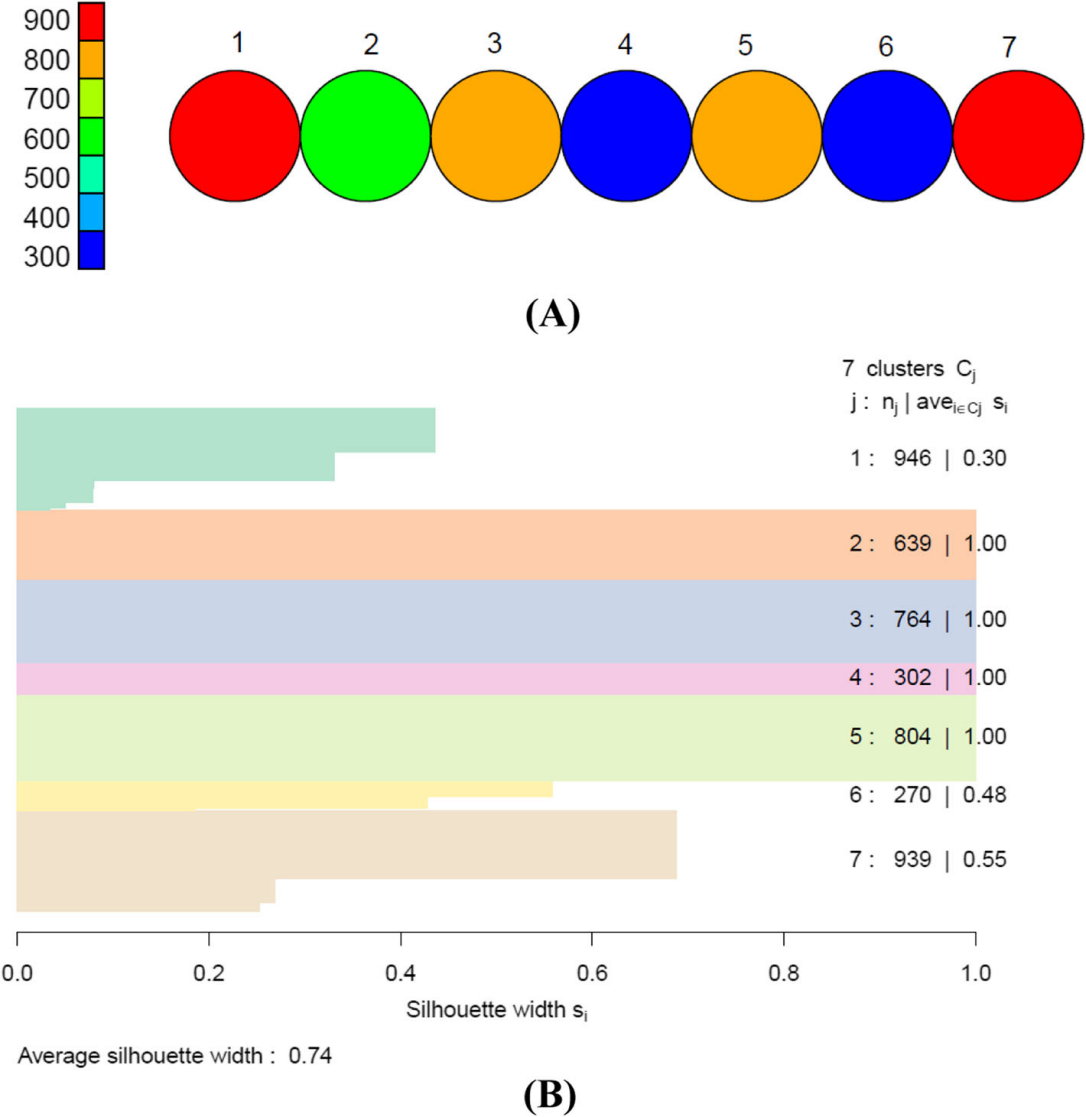
**a** Overall clusters identified by SOM in women with size of 7 × 1. Each circle represents one cluster. Each cluster contains 300–900 subjects who are extremely similar to each other regarding to four MRFs. **b** The silhouette width of each cluster and average silhouette width of all clusters. SOM: self-organizing maps; MRFs: metabolic risk factors

We obtained seven apparent clusters in both genders by the SOM based on hexagonal topology with size 7 × 1. The average silhouette width was 0.61 and 0.74 for men and women, respectively, indicating a strong and reasonable clustering structure (Figs. 3 and 4).

### Description of the clusters in men and women

The baseline characteristics of the clusters in men are presented in Table 2. Cluster 1, including 17.1% of men and mean age of 53.5 years, had the highest mean number of MRFs (3.4). All population (100%) in this cluster had HTC and HFPG. Also, 54 and 34.5% of men in this cluster had four and three MRFs, respectively. Cluster 7, with the lowest mean number (0.4) of MRFs, described 21.4% of men with mean age of 43.6 years. About 41.6% of population in this cluster had only one MRF including HTC. The highest and lowest incidence rates of cancer (5.1 and 0.6 per 1000 person years) were found in cluster 3 and cluster 5, respectively.

**Table 2:** Baseline characteristics of the clusters in male population, Tehran Lipid and Glucose Study (1999–2014)

Variables	Cluster 1 (***n*** = 674)	Cluster 2 (***n*** = 411)	Cluster 3 (***n*** = 498)	Cluster 4 (***n*** = 672)	Cluster 5 (***n*** = 456)	Cluster 6 (***n*** = 375)	Cluster 7 (***n*** = 843)	*P value
**Age (years)**	53.5(12.4)	52.3(13.1)	52.4(14.2)	51.0(13.4)	42.4(9.8)	40.8(9.3)	43.6(11.5)	< 0.001
**Education**								
Level 1 (0–8 years)	520(77.2)	324(78.8)	373(74.9)	507(75.4)	354(77.6)	291(77.6)	634(75.2)	< 0.001
Level 2 (9–12 years)	98(14.5)	54(13.1)	79(15.9)	113(16.8)	89(19.5)	78(20.8)	173(20.5)	
Level 3 (> 12 years)	56(8.3)	33(8.1)	46(9.2)	52(7.8)	13(2.9)	6(1.6)	36(4.3)	
**Physical activity level**								
Low physical activity	490(72.7)	287(69.8)	326(65.5)	498(74.1)	343(75.2)	279(74.4)	627(74.4)	0.005
**Smoking**								
Never	291(43.2)	173(42.1)	207(41.6)	296(44.0)	161(35.3)	134(35.7)	256 (30.3)	< 0.001
Past	133(19.7)	77(18.7)	89(17.9)	125(18.6)	68(14.9)	48(12.8)	111(13.2)	
Passive	73 (10.8)	50(12.2)	59(11.8)	102(15.2)	66(14.5)	57(15.2)	95 (11.3)	
Current	177(26.3)	111(27.0)	143(28.7)	149(22.2)	161(35.3)	136(36.3)	381(45.2)	
**BMI (kg/m**^**2**^**)**	27.6(3.9)	26.7(4.1)	26.2(3.7)	27.1(3.8)	28.2(2.6)	27.9(2.2)	22.1(2.1)	< 0.001
**FPG (mmol/L)**	7.4(2.7)	7.1(2.3)	4.9(0.3)	4.9(0.3)	4.9(0.4)	4.9(0.3)	4.8(0.3)	< 0.001
**TC (mmol/L)**	6.2(0.8)	4.4(0.5)	4.4(0.5)	6.1(0.8)	6.0(0.8)	4.4(0.5)	4.9(1.0)	< 0.001
**SBP (mmHg)**	130.2(21.2)	127.8(20.7)	135.8(16.3)	134.0(14.7)	109.2(6.7)	108.5(7.5)	106.4(8.2)	< 0.001
**Mean number of risk factors**	3.4(0.7)	2.2(0.7)	1.6(0.5)	2.7(0.5)	2.0(0.0)	1.0(0.0)	0.4(0.4)	< 0.001
**HBMI**	511(75.8)	277(67.4)	302(60.6)	486(72.3)	456(100)	375(100)	0(0.0)	< 0.001
**HSBP**	450(66.8)	256(62.3)	498(100)	672(100)	0(0.0)	0(0.0)	0(0.0)	< 0.001
**HTC**	674(100)	0 (0.0)	0 (0.0)	672 (100)	456 (100)	0(0.0)	351(41.6)	< 0.001
**HFPG**	674(100)	411(100)	0(0.0)	0(0.0)	0(0.0)	0(0.0)	0(0.0)	< 0.001
**Number of events**	29	16	30	27	4	8	18	
**Incident rate of cancers (CI), per 1000 person years**	3.6(2.5–5.2)	3.4(2.0–5.5)	5.1(3.6–7.3)	3.2(2.2–4.7)	0.6(0.2–1.8)	1.7(0.8–3.5)	1.7(1.1–2.7)	

Figures are presented as mean (standard deviation) for continuous variables, and number (percentage) for categorical variables

*CI* confidence interval, *HBMI* high body mass index, *HFPG* high fasting plasma glucose, *HTC* high total cholesterol, *HDL-C* high systolic blood pressure

*P values are based on ANOVA test for continuous variables and chi-square test for categorical variables

Table 3 presents the characteristics of the clusters of the MRFs in women. About 20% of females belonged to cluster 7 with mean age of 53.6 years, of whom 33% had four, and 67% had three MRFs. All women (100%) in cluster 7 had HFPG and HTC, and most of them (90%) had HBMI. Cluster 1 included a group of women with the lowest mean number of MRFs (0.8). This cluster contained 20.2% of women with mean age of 42.4 years, of whom 43% had no MRFS.

**Table 3:** Baseline characteristics of the clusters in female population, Tehran Lipid and Glucose Study (1999–2014)

Variables	Cluster 1 (***n*** = 946)	Cluster 2 (***n*** = 639)	Cluster 3 (***n*** = 764)	Cluster 4 (***n*** = 302)	Cluster 5 (***n*** = 804)	Cluster 6 (***n*** = 270)	Cluster 7 (***n*** = 939)	***P*** value
**Age (years)**	42.4(12.1)	38.5(6.6)	43.3(9.8)	46.7(10.5)	52.0(10.9)	48.2(10.9)	53.6(10.4)	<0.001
**Education**								
Level 1 (0–8 years)	724(76.5)	542(84.8)	637(83.4)	233(77.2)	580(72.1)	203(75.2)	649(69.1)	<0.001
Level 2 (9–11 years)	128(13.6)	73(11.4)	66(8.6)	23(7.6)	24(3.0)	10(3.7)	24(2.6)	
Level 3 (> 12 years)	94(9.9)	24(3.8)	61(8.0)	46 (15.2)	200 (24.9)	57(21.1)	266(28.3)	
**Physical activity level**								
Low physical activity	681(72.0)	425(66.5)	536(70.2)	205(67.9)	580(72.1)	186(68.9)	700(74.5)	0.019
**Smoking**								
Never	710(75.1)	468(73.2)	541(70.8)	228(75.5)	631(78.5)	177(65.6)	722(76.9)	<0.001
Past	14(1.5)	7(1.1)	14(1.9)	2(0.7)	31(3.8)	13(4.8)	32(3.4)	
Passive	168(17.7)	119(18.7)	169(22.1)	58(19.2)	118(14.7)	63(23.3)	153(16.3)	
Current	54(5.7)	45(7.0)	40(5.2)	14(4.6)	24(3.0)	17(6.3)	32(3.4)	
**BMI (kg/m**^**2**^**)**	22.6(1.8)	29.2(3.2)	29.6(3.4)	30.3(4.1)	30.6(3.8)	30.4(4.5)	30.4(4.7)	<0.001
**FPG (mmol/L)**	5.0(1.3)	4.8(0.3)	4.8(.3)	0.3(2.4)	4.9(0.4)	7.1(2.3)	7.9(3.2)	<0.001
**TC (mmol/L)**	5.1(1.1)	4.4(0.5)	6.2(0.9)	4.6(0.4)	6.4(0.9)	4.6(0.4)	6.6(1.1)	<0.001
**SBP (mmHg)**	111.2(16.5)	106.5(7.5)	108.1(7.7)	132.7(13.0)	137.1(16.5)	125.3(17.5)	135.1(22.5)	<0.001
**Mean number of risk factors**	0.8(0.7)	1.0(0.0)	2.0 (0.0)	2.0 (0.0)	3.0 (0.0)	2.5(0.5)	3.6(0.4)	<0.001
**HBMI**	(0.0)	639(100)	764(100)	302(100)	804(100)	250(92.6)	850(90.5)	<0.001
**HSBP**	210(22.2)	0(0.0)	0 (0.0)	302(100)	804(100)	161(59.6)	718(76.5)	<0.001
**HTC**	436(46.1)	0(0.0)	764(100)	0(0.0)	804(100)	0(0.0)	939(100)	<0.001
**HFPG**	73(7.7)	0(0.0)	0(0.0)	0(0.0)	0(0.0)	270(100)	939(100)	<0.001
**Number of events**	31	7	20	8	27	16	24	
**Incident rate of cancers (CI), per 1000 person years**	2.6(1.8–3.7)	0.8(0.4–1.8)	2.1(1.3–3.2)	2.1(1.0–4.2)	2.6(1.8–3.8)	5.0 (3.0–8.2)	2.1(1.4–3.1)	

Figures are presented as mean (standard deviation) for continuous variables, and number (percentage) for categorical variables

*CI* confidence interval, *HBMI* high body mass index, *HFPG* high fasting plasma glucose, *HTC* high total cholesterol, *HDL-C* high systolic blood pressure

*P values are based on ANOVA test for continuous variables and chi-square test for categorical variables

### Sociodemographic predictors of cluster membership

According to multinomial logistic regression models, age, smoking, and physical activity were significant predictors for cluster membership in men (Table 4). Considering cluster 7 (relatively normal group) as reference, a group of physically inactive men were less likely than physically active men to be in the cluster 3 [odds ratio (OR) 0.69 (95% CI 0.54–0.89)]. Older men were more likely than younger men to be in clusters 1, 2, 3, and 4 compared with cluster 7 [1.06(1.05–1.07), 1.05(1.04–1.06), 1.06(1.04–1.06), and 1.05(1.04–1.06), respectively]. Also, in men, current smokers were less likely than never smokers to be in the clusters 1, 2, 3, 4, 5, and 6 compared with the cluster 7 [0.52(0.41–0.65), 0.51(0.39–0.67), 0.56(0.43–0.73), 0.37(0.29–0.48), 0.63(0.49–0.81), and 0.62(0.47–0.81), respectively] (Table 4).

**Table 4:** Associations between individuals' characteristics and clusters membership in men, Tehran Lipid and Glucose Study (1999–2014)

	Cluster 1		Cluster 2		Cluster 3		Cluster 4		Cluster 5		Cluster 6	
Variables	OR(95%CI)	***P*** value	OR(95%CI)	***P*** value	OR(95%CI)	***P*** value	OR(95%CI)	***P*** value	OR(95%CI)	***P*** value	OR(95%CI)	***P*** value
**Age (years)**	1.06(1.05–1.07)	< 0.001	1.05(1.04–1.06)	< 0.001	1.06(1.04–1.06)	< 0.001	1.05(1.04–1.06)	< 0.001	0.98(0.97–1.00)	0.059	0.97(0.96–0.98)	< 0.001
**Education**												
Level 3 (> 12 years)	**Reference**											
Level 1 (0–8 years)	1.56(0.97–2.49)	0.063	1.44(0.85–2.44)	0.169	1.17(0.71–1.91)	0.530	1.34(0.83–2.15)	0.225	1.37(0.70–2.71)	0.354	1.92(0.77–4.7)	0.158
Level 2 (9–12 years)	1.31(0.77–2.24)	0.313	1.03(0.55–1.88)	0.932	1.06(0.60–1.85)	0.829	1.20(0.70–2.04)	0.497	1.15(0.56–2.38)	0.693	1.65(0.64–4.22)	0.296
**Physical activity level**												
High	**Reference**											
Low	1.00(0.78–1.27)	0.995	0.86(0.65–1.12)	0.256	0.69(0.54–0.89)	0.004	1.05(0.83–1.34)	0.672	1.05(0.80–1.37)	0.714	0.99(0.75–1.32)	0.989
**Smoking**												
Never	**Reference**											
Past	0.89(0.64–1.29)	0.625	0.88(0.60–1.10)	0.559	0.86(0.59–1.25)	0.446	0.85(0.591.18)	0.315	0.99(0.58–1.34)	0.559	0.88(0.71–1.44)	0.922
Passive	0.93(0.65–1.33)	0.712	1.03(0.69–1.55)	0.861	1.04(0.71–1.53)	0.828	1.16(0.83–1.62)	0.374	1.05(0.72–1.53)	0.770	1.05(0.71–1.56)	0.780
Current	0.51(0.41–0.65)	< 0.001	0.52(0.39–0.67)	< 0.001	0.57(0.43–0.73)	< 0.001	0.39(0.29–0.48)	< 0.001	0.64(0.49–0.81)	< 0.001	0.63(0.47–0.81)	0.001

Results obtained from the multinomial logistic regression. Cluster 7 with the lowest mean number of MRFs (Table 2) was considered as reference group in multinomial regression analysis

*MRFS* metabolic risk factors, *OR* odds ratio, *CI* confidence interval

*P values are based on statistical significance of OR

In women, age, physical activity, educational level, and smoking status were predictors of cluster membership at baseline (Table 5). Older women were more likely than younger to be in the clusters 3, 4, 5, 6, and 7 compared with the cluster 1 (relatively normal group) as reference group [1.01(1.00–1.02), 1.04(1.02–1.05), 1.08(1.07–1.09), 1.04(1.03–1.06), and 1.09(1.081.10), respectively]. Results also showed that women with a low educational level were more likely than highly educated participants to be in cluster 3 compared with cluster 1 [1.63(1.12–2.38)]. On the contrary, highly educated women were less likely than low educated women to be in clusters 5, 6, and 7, compared with cluster 1 [0.40(0.23–0.69), 0.30(0.14–0.66), 0.38(0.22–0.65)]. Further, women who currently smoked were less likely than never smokers to be in cluster 5, compared with cluster 1 [0.54(0.33–0.91)] (Table 5). Also, among women, passive smoking was associated with increased chance of being in clusters 3 and 6 [1.33(1.04–1.70) and 1.63(1.16–2.29), respectively].

**Table 5:** Associations between individuals' characteristics and clusters membership in women, Tehran Lipid and Glucose Study (1999–2014)

Variables	Cluster 2		Cluster 3		Cluster 4		Cluster 5		Cluster 6		Cluster 7	
	OR(95%CI)	***P*** value	OR(95%CI)	***P*** value	OR(95%CI)	***P*** value	OR(95%CI)	***P*** value	OR(95%CI)	***P*** value	OR(95%CI)	***P*** value
**Age (years)**	0.94(0.93–0.96)	<0.001	1.01(1.00–1.02)	0.021	1.04(1.02–1.05)	<0.001	1.08(1.07–1.09)	<0.001	1.04(1.03–1.06)	<0.001	1.09(1.08–1.10)	<0.001
**Education**												
Level 3 (> 12 years)	**Reference**											
Level 1 (0–8 years)	1.40(0.85–2.30)	0.186	1.65(1.13–2.41)	0.009	1.12(0.72–1.73)	0.579	1.16(0.85–1.59)	0.351	0.92(0.59–1.41)	0.713	1.14(0.84–1.54)	0.388
Level 2 (9–12 years)	0.87(0.48–1.56)	0.648	1.03(0.61–1.63)	0.890	0.74(0.39–1.38)	0.365	0.40(0.23–0.69)	0.001	0.32(0.14–0.70)	0.003	0.38(0.22–0.65)	0.001
**Physical activity level**												
High	**Reference**											
Low	0.76(0.61–0.95)	0.017	0.90(0.73–1.12)	0.372	0.79(0.60–1.05)	0.115	0.92(0.74–1.15)	0.511	0.81(0.60–1.09)	0.172	1.03(0.83–1.28)	0.758
**Smoking**												
Never	**Reference**											
Past	1.08(0.42–2.74)	0.869	1.26(0.56–2.54)	0.638	0.31(0.07–1.40)	0.129	1.31(0.67–2.56)	0.424	2.37(0.97–4.73)	0.057	1.06(0.53–2.04)	0.898
Passive	0.99(0.76–1.29)	0.955	1.33(1.04–1.70)	0.019	1.15(0.82–1.62)	0.393	0.93(0.71–1.22)	0.630	1.63(1.16–2.29)	0.004	1.10(0.85–1.42)	0.468
Current	1.25(0.82–1.89)	0.290	0.97(0.59–1.39)	0.671	0.82(0.43–1.46)	0.469	0.54(0.33–0.91)	0.020	1.30(0.66–2.07)	0.587	0.64(0.40–1.02)	0.063

Results obtained from the multinomial logistic regression. Cluster 1 with the lowest mean number of MRFs (Table 3) was considered as reference group in multinomial regression analysis

*MRFS* metabolic risk factors, *OR* odds ratio, *CI* confidence interval

*P values are based on statistical significance of OR

### Cluster membership and incident cancers

Among men, individuals in cluster 5 had the lowest incidence rate of cancer (Table 2) during median follow-up of 13.9 years. The confounders adjusted risk was more than three times higher in cluster 3 (hazard ratio (HR) 3.56, 95% CI 1.23–10.28) compared with cluster 5 (Table 6).

**Table 6:** Cluster membership in relation to cancer incidence in Iranian population in the Tehran Lipid and Glucose Study (1999–2014)

	Model 1^**a**^			Model 2^**b**^		
	HR	(95% CI)	***P*** value	HR	(95% CI)	***P*** value
**Men**						
Cluster 5	**Reference**	–	–	**Reference**	–	–
Cluster 1	5.38	(1.89–15.33)	0.002	2.56	(0.89–7.39)	0.080
Cluster 2	5.17	(1.73–15.49)	0.003	2.65	(0.87–8.03)	0.083
Cluster 3	7.75	(2.73–22.01)	0.003	3.56	(1.23–10.28)	0.018
Cluster 4	4.76	(1.66–13.61)	0.001	2.46	(0.85–7.11)	0.096
Cluster 6	2.60	(0.78–8.63)	0.118	3.05	(0.91–10.14)	0.068
Cluster 7	2.58	(0.87–7.65)	0.085	2.23	(0.75–6.62)	0.146
**Women**						
Cluster 2	**Reference**	–	–	**Reference**	–	–
Cluster 1	2.92	(1.28–6.63)	0.010	2.23	(0.97–5.13)	0.057
Cluster 3	2.32	(0.98–5.49)	0.054	1.78	(0.75–4.25)	0.190
Cluster 4	2.36	(0.85–6.51)	0.096	1.54	(0.55–4.33)	0.404
Cluster 5	2.98	(1.30–6.86)	0.009	1.53	(0.64–3.64)	0.330
Cluster 6	5.78	(2.38–14.07)	<0.001	3.63	(1.46–8.99)	0.005
Cluster 7	2.42	(1.04–5.61)	0.039	1.20	(0.49–2.88)	0.683

^a^Model was unadjusted

^b^Model was adjusted for age, smoking status, and educational and physical activity levels

In the Cox regression models, cluster 5 and cluster 2 in men and women, respectively, with the lowest incidence rates of cancer (Tables 2 and 3) were considered as reference groups

*CI* confidence interval, *HR* hazard ratio

Among women, subjects in cluster 2 had the lowest incidence rate of cancer (Table 3) during median follow-up of 14.1 years. Women in cluster 6 had nearly four times higher adjusted risk of cancer than did women in cluster 2 (3.63, 1.46–8.99) (Table 6).

Finally, we repeated the analysis while considering the clusters with highest incidence rate of cancer as reference groups. Accordingly, among men, only cluster 5 showed a lower risk of cancer (0.28, 0.09–0.80) compared with cluster 3 as reference. However, among women, all clusters except cluster 1 had lower risk of cancer than did women in cluster 6 as reference (Supplementary Table 1).

## Discussion

In this longitudinal study of 8593 Iranian adults with a median of 14 years follow-up, the prevalence and clustering of four major MRFs were identified. Moreover, sociodemographic determinants related to cluster membership were identified. We also investigated how different clusters of MRFs were associated with increases or decreases in cancer development. In both genders, seven distinct clusters of four MRFs were identified by SOM. These clusters differed substantially from each other in terms of total number of risk factors, the associations between identified clusters with four sociodemographic factors (age, educational level, physical activity level, and smoking status), and incidence of composite of cancer types. Among men, cluster 3 including those with 100% HSBP had significantly greater risk of incident cancer compared with cluster 5 as the reference group. In females, cluster 6 including individuals with 100% HFPG had significantly higher risk of cancer than cluster 2 as the reference group.

The present study found that the presence of four MRFs, individually or in combination, is highly prevalent in Iranian adults, as we have previously shown [7]. About 88 and 91% of men and women, respectively, had at least one MRF, and 32% of men and 40% of women were found to engage in three or four MRFs.

A large number of studies have previously examined the association between MRFs and cancer incidence. However, they have focused on only one MRF [24, 25] or pre-defined constellation of factors such as metabolic syndrome [8]. In contrast, our study extracted different patterns of MRFs and their effect on cancer risk.

### Clustering patterns in men

Among male participants, a relatively healthy subgroup (cluster 7) with the lowest number of MRFs was identified in which 41.6% of subjects had only one MRF. In particular, we identified two unhealthy subgroups (clusters 1 and 4), of whom 100% had at least two MRFs. Association analysis showed that each 1-year increment in age was associated with about 5% increase in chance of being in clusters 1 to 4, with high number of MRFs. It is assumed that aging is the result of the accumulation of multiple forms of damage and pathology in different tissues [26].

Surprisingly, we found that smoking decreased the chance of being in unhealthy clusters (clusters 1 to 6) compared to healthy cluster (cluster 7). In fact, our results suggest that smoking decreases the aggregation of MRFs. Our results confirm the findings of previous studies that suggest that smoking has a protective effect against some MRFs [27]. The inverse association between smoking and clustering of MRFs might be attributable to diminished appetite, rise in metabolic rate, and as a result, lower measures of abdominal obesity and blood pressure among smokers [27].

Recent studies showed a convincing association between metabolic syndrome, as aggregation of three or more metabolic disorders, and certain types of cancer, including prostate [28] and breast [29]. Interestingly, we did not find a clear relationship between the number of MRFs and cancer risk in men. For example, we found the highest incidence rate of cancer in cluster 3, although they had fewer MRFs than clusters 1 and 4. Also, individuals in cluster 7 had the lowest number of MRFs; however, they had a higher incidence rate of cancer compared with cluster 5 (1.7 vs. 0.6). Multivariate Cox regression analyses revealed that cluster 3 (with the highest incidence rate) had more than 3.5-fold the adjusted cancer risk of cluster 5 (with the lowest incidence rate). In complementary analysis (Supplementary Table 1), individuals in cluster 5 had 72% lower risk of cancer after adjustment for confounders compared with cluster 3. Several interesting findings emerge from the patterns identified among men. Firstly, comparison of patterns in clusters 3, 5, 6, and 7 suggests that combined effects of HBMI and HTC (cluster 5) has a more protective effect against cancer risk than individual effects of these two risk factors (clusters 6 and 7). Many studies have investigated the individual effects of BMI and TC on the risk of incident cancer, but research findings have been inconsistent*.* In a large pooled cohort of Australian adults, BMI was associated with the development of overall, colorectal, and obesity-related cancers in men [30]. Also, several studies have documented a positive association between BMI and risk of prostate cancer [31]. A Chinese cohort study, conducted on 133273 subjects, found a significant association between BMI and risk for cancer incidence. Among men, underweight (BMI < 18.5 kg/m^2^) increased the risk of gastric and liver cancer, and obesity (BMI ≥ 28.0 kg/m^2^) increased the risk of colon cancer. However, overweight (BMI 24–28 kg/m^2^) showed a protective role in lung and bladder cancer incidence in males [32]. Several biological mechanisms have been suggested for the association between HBMI and risk of various cancers. They include obesity-related hormones, growth factors, modulation of energy balance and calorie restriction, multiple signaling pathways, and inflammatory processes affecting cancer cell promotion and progression [33].

In recent years, HTC has been linked to the development of several different cancers although the results are inconsistent. A number of studies have reported a positive association between TC and cancers [24, 34]. However, others found lower overall or site-specific cancer incidence in people with high TC levels [25, 35]. A Korean cohort study showed that a high TC level (≥ 240 mg/dL) was negatively associated with risk of liver, stomach cancer in both men and women, and lung cancer in men [25]. It has been suggested that the observed inverse associations between TC levels and cancer risk is an effect of preclinical cancer or disease due to an increased uptake of cholesterol by tumor cells rather than reflecting a true causal relationship on cholesterol levels [36]. In the present study, we found that co-occurrence of HBMI and HTC put men to a lower risk of cancer compared with the occurrence of the individual factors alone.

Another interesting finding emerges from the comparison of four clusters 1, 2, 3, and 4 with three other clusters (5, 6, and 7); the highest incidence of cancer was observed in clusters at which all or most of the subjects had HSBP (clusters 1 to 4). Also the identified patterns in clusters 3 and 4 suggest that HTC may modify the adverse effect of HSBP, because the risks of cancer were not significantly different between clusters 3 and 4, despite the 100% prevalence of HSBP and HTC in cluster 4. In some studies, arterial hypertension was associated with a higher risk of colorectal [37], prostate [8] cancer, and malignant melanoma [38]. Also, the arterial hypertension was found to be closely linked with renal cell cancer development [39]. There are many uncertainties regarding a possible relation between hypertension and cancer, mainly concerning cancer site specificity, sex, age, and duration of the disease, and also complex interactions with other factors, such as smoking, BMI, diabetes, alcohol, and diet [40]. According to our analysis, the combination of HSBP and HBMI could be conceptualized as a very high risk pattern for overall cancer incidence among Iranian men. From a public health perspective, these results are important due to the high prevalence of hypertension and obesity among Iranian population [7, 41].

### Clustering patterns in women

Among females, cluster 1 was found to be relatively healthier than the others, with the lowest mean number of MRFs (0.8). About 43% of women in this cluster had no MRFs. Furthermore, we found two clusters with multiple MRFs (clusters 5 and 7), of whom 100% had at least two MRFs. Association analysis showed a positive relation between aging and clustering of MRFs, similar association was found in men. In addition, moderate education decreased the chance of being in unhealthy clusters (clusters 5, 6, and 7) compared with high education. This finding may be attributable to sedentary lifestyle among highly educated women. One study reported that those with high education had lower total physical activity than those with moderate education [42]. Interestingly, we found that smoking decreased the chance of being in cluster 5, in which all individuals had HBMI, HSBP, and HTC. This suggests the protective effect of smoking on some MRFs [27], as we discussed in the previous section. However, passive smoking increased the chance of being in clusters 3 and 6 with a relatively large number of MRFs. A recent meta-analysis reported a positive association between passive smoking and some cardiometabolic risk factors such as BMI, FPG, and LDL-C which vary with age [43].

In women, the highest incidence rate of cancer was observed in cluster 6, although the number of MRFs was relatively smaller than clusters 5 and 7. Thus, unlike some previous studies [29], our finding did not show a clear relationship between the number of MRFs and cancer risk in women. The results of multivariate Cox regression showed that cluster 6 (with the highest incidence rate) had about 3.6-fold increased risk for cancer compared with cluster 2 (with the lowest incidence rate). Furthermore, cluster 1 had about 2.2-fold increased risk compared with cluster 2 (marginally significant). In complementary analysis (Supplementary Table 1), we found that all clusters, except cluster 1, had significantly lower adjusted risk of cancer compared with cluster 6. Some important conclusions emerge from these findings; firstly, healthy overweight or obese women (cluster 2) showed the lowest overall cancer risk.

Evidence has suggested that BMI is an important predictor of cancer risk [44]: a population-based cohort study of 5.24 million UK adults showed associations between increased BMI and certain types of cancer [45]. In a meta-analysis of 221 datasets, positive associations were reported between HBMI and cancers of the esophagus, thyroid, colon, kidneys, endometrium, and gallbladder; in contrast, increased BMI was negatively associated with lung cancer [46].

The relation between BMI and cancer are complex and are not yet fully understood. For example, some studies have shown that increased BMI is associated with an increased risk of breast cancer in women after menopause [47]; however, a meta-analysis showed that BMI had no significant effect on the incidence of breast cancer during the premenopausal period [48]. In our study, the lowest incidence of cancer in cluster 2 may be due to the age, as this cluster was the youngest group (mean age of 38 years) among 7 clusters.

Very few studies have examined the effects of various combinations of BMI and other MRFs on cancer risk. Our study showed that healthy overweight/obese women (cluster 2) had the lowest risk for incidence of cancer but the risk significantly increased only when HFPG is added to HBMI (clusters 6 and 1). All individuals (100%) and 7.7% of individuals in cluster 6 and cluster 1, respectively, had HFPG; in contrast, nobody had HFPG in cluster 2, reinforcing the positive association between HFPG and cancer risk.

While many observational studies suggest that people with pre-diabetes and diabetes are at a significantly higher risk of some types of cancer [49], but the links between them are incompletely understood. A prospective cohort study of 1,298,385 Koreans (468,615 women) aged 30 to 95 years reported significant positive associations between fasting serum glucose and cancers of the liver and cervix in women [50]. One cohort study in Scotland showed significantly increased risks for pancreatic, liver, and colon cancer in all population, while no significant association was found between diabetes and overall cancer [51]. In conclusion, pre-diabetes/diabetes and cancer have a complex relationship that requires more clinical attention and better-designed studies.

Interestingly, all individuals in cluster 7 had also HFPG; however, no statistically significant differences in risk of cancer were observed between cluster 7 and cluster 2. Unlike cluster 2, all individuals in cluster 7 had HTC which suggests the protective role of HTC against cancer risk, as we have discussed in the previous section.

In sum, we observed the sex-specific risk patterns of overall cancers. It was found that combination of HBMI and HFPG put women at the highest risk for overall cancer. A recent meta-analysis [52] found that diabetic women had a 27% higher risk of cancer compared with non-diabetic women, while men with diabetes had a 19% higher risk of cancer compared with men without diabetes. Also, women with diabetes had a higher risk for most cancers than men with diabetes. The possible mechanisms include as follows: (1) women often spend longer duration than men in the pre-diabetic stage, (2) diabetic women have poor glycemic control compared with men with diabetes, (3) women are often undertreated or not getting the same level of treatment as men. Therefore, women with diabetes may be at greater risk of developing cancer than men due to carcinogenic effects of hyperglycemia and DNA damage [53]. Interestingly, we observed that the presence of two MRFs including HSBP and HBMI significantly increased the risk of total cancer in men. Observational studies have shown inconsistent results for the association between blood pressure and cancer risk. A large study using data from 7 cohort studies investigated the association among 577,799 adults with a mean age of 44 years. The results showed a small increased overall cancer risk only in men [54]. However, a recent meta-analysis showed the positive associations between hypertension and several cancers in both genders such as kidney, colorectal, and breast cancer [55]. Although the association of each isolated metabolic factors with cancer incidence has been investigated in a large number of studies, to our knowledge, no study has been conducted to evaluate the relationship between clusters of MRFs and total cancer in adult population. Therefore, the relation between this risk pattern (HSBP and HBMI) and cancer is poorly understood and needs further carefully designed studies taking into account different combinations of MRFs.

Several limitations should be noted. First, MRFs were measured only once at cohort entry, so we were unable to assess changes over time. Second, due to the small number of cancer cases, we were unable to stratify results by cancer site. Third, the Iranian background of study participants may limit the generalizability of our findings to more diverse ethnic groups of population. Strengths of our study include its long duration of follow-up and population-based sample. Also, a comprehensive physical exam and questionnaire were completed at cohort entry, and complete and reliable outcome data obtained through the outcome committee team. This is the first study that identified multiple clusters of MRFs using SOM in a well-characterized cohort of Iranian population.

## Conclusion

Clustering of MRFs is common in Iran. The majority of men had more than one metabolic risk factor. Multiple modifiable factors such as educational level, physical activity level, and smoking are responsible for the clustering of MRFs. In general, a gradient between the number of MRFs and cancer risk was not observed in both men and women. Instead, some combinations of four MRFs were significantly associated with an increased risk of overall cancer. Our study shows that co-occurrence of HBMI and HFPG in women, and HSBP and HBMI in men are powerful indicators of overall cancer. However, HBMI in combination with HTC has a protective effect against cancer development in both genders. These findings suggest that the combined information from a few variables related to cancer development is superior to measurement of only one metabolic risk factor. The cumulative and clustered nature of MRFs helps identify potential mechanisms of cancer and modifiable factors that can serve as important ways for intervention initiatives.

### Perspectives and significance

This study provides new evidence that the presence of a MRF alone is not a reliable predictor for cancer risk. Instead, clusters of MRF signal an opportunity for screening and primary prevention programs. From a public health perspective, our results have important implication because metabolic risk factors are highly prevalent in our country with inadequate control.

## Supplementary information

**Additional file 1: Supplementary Table 1.** Cluster membership in relation to cancer incidence in Iranian population in the Tehran Lipid and Glucose Study (1999-2014)

## Data Availability

The datasets analyzed during the current study are available from the corresponding author on reasonable request.
