# Supplementary material for: Sex-specific clustering of metabolic risk factors and cancer risk: a longitudinal study in Iran
Source: Biol Sex Differ. 2020 Apr 25;11:21. doi: 10.1186/s13293-020-00296-6 (PMC7183600; doi:10.1186/s13293-020-00296-6)
Supplement: Supplementary file 1 — Additional file 1: Supplementary Table 1. Cluster membership in relation to cancer incidence in Iranian population in the Tehran Lipid and Glucose Study (1999-2014) [file 13293_2020_296_MOESM1_ESM.docx]

**Supplementary Table 1** Cluster membership in relation to cancer incidence in Iranian population in the Tehran Lipid and Glucose Study (1999-2014)

|  | **Model 1^a^** | | | **Model 2^b^** | | |
| --- | --- | --- | --- | --- | --- | --- |
|  | HR | (95% CI) | **P-value** | HR | (95% CI) | **P-value** |
| **Men** | | | | | | |
| Cluster 3 | **Reference** | **--** | **--** | **Reference** | **--** | **--** |
| Cluster 1 | 0.69 | (0.41-1.15) | 0.162 | 0.72 | (0.43-1.20) | 0.209 |
| Cluster 2 | 0.66 | (0.36-1.22) | 0.192 | 0.74 | (0.40-1.36) | 0.342 |
| Cluster 4 | 0.61 | (0.36-1.03) | 0.066 | 0.69 | (0.40-1.16) | 0.164 |
| Cluster 5 | 0.12 | (0.04-0.36) | <0.001 | 0.28 | (0.09-0.80) | 0.018 |
| Cluster 6 | 0.33 | (0.15-0.73) | 0.006 | 0.85 | (0.37-1.92) | 0.707 |
| Cluster 7 | 0.33 | (0.18-0.59) | <0.001 | 0.62 | (0.34-1.14) | 0.129 |
| **Women** | | | | | | |
| Cluster 6 | **Reference** | **--** | **--** | **Reference** | **--** | **--** |
| Cluster 1 | 0.50 | (0.27-0.92) | 0.026 | 0.61 | (0.33-1.13) | 0.118 |
| Cluster 2 | 0.17 | (0.07-0.41) | <0.001 | 0.27 | (0.11-0.68) | 0.005 |
| Cluster 3 | 0.40 | (0.20-0.77) | 0.006 | 0.49 | (0.25-0.95) | 0.036 |
| Cluster 4 | 0.41 | (0.17-0.95) | 0.038 | 0.42 | (0.18-0.99) | 0.049 |
| Cluster 5 | 0.51 | (0.27-0.95) | 0.036 | 0.42 | (0.22-0.78) | 0.006 |
| Cluster 7 | 0.42 | (0.22-0.78) | 0.006 | 0.33 | (0.17-0.62) | <0.001 |
| ^a^ Model was unadjusted  ^b^ Model was adjusted for age, smoking status, educational and physical activity levels  In the Cox regression models cluster 3 and cluster 6 in men and women, respectively, with the highest incidence rates of cancer (Table 1 and 2) were considered as reference groups.  **CI:** confidence interval; **HR:** hazard ratio | | | | | | |
